# MT5-MMP Promotes Alzheimer’s Pathogenesis in the Frontal Cortex of 5xFAD Mice and APP Trafficking *in vitro*

**DOI:** 10.3389/fnmol.2016.00163

**Published:** 2017-01-10

**Authors:** Kévin Baranger, Amandine E. Bonnet, Stéphane D. Girard, Jean-Michel Paumier, Laura García-González, Wejdane Elmanaa, Anne Bernard, Eliane Charrat, Delphine Stephan, Charlotte Bauer, Katrin Moschke, Stefan F. Lichtenthaler, François S. Roman, Frédéric Checler, Michel Khrestchatisky, Santiago Rivera

**Affiliations:** ^1^Aix Marseille Université, CNRS, NICN UMR 7259Marseille, France; ^2^Université Côte d’Azur, INSERM, CNRS, IPMC, Laboratory of excellence DistALZ, Sophia-AntipolisValbonne, France; ^3^German Center for Neurodegenerative Diseases (DZNE)Munich, Germany; ^4^Neuroproteomics, Klinikum rechts der Isar, and Institute for Advanced Study, Technische Universität München (TUM)Munich, Germany; ^5^Munich Cluster for Systems Neurology (SyNergy)Munich, Germany

**Keywords:** matrix metalloproteinase, amyloid beta peptide, amyloid precursor protein, learning and memory, endosomes, transgenic mice

## Abstract

We previously reported that deficiency of membrane-type five matrix metalloproteinase (MT5-MMP) prevents amyloid pathology in the cortex and hippocampus of 5xFAD mice, and ameliorates the functional outcome. We have now investigated whether the integrity of another important area affected in Alzheimer’s disease (AD), the frontal cortex, was also preserved upon MT5-MMP deficiency in 4-month old mice at prodromal stages of the pathology. We used the olfactory H-maze (OHM) to show that learning impairment associated with dysfunctions of the frontal cortex in 5xFAD was prevented in bigenic 5xFAD/MT5-MMP^−/−^ mice. The latter exhibited concomitant drastic reductions of amyloid beta peptide (Aβ) assemblies (soluble, oligomeric and fibrillary) and its immediate precursor, C99. Simultaneously, astrocyte reactivity and tumor necrosis factor alpha (TNF-α) levels were also lowered. Moreover, MT5-MMP deficiency induced a decrease in N-terminal soluble fragments of amyloid precursor protein (APP), including soluble APPα (sAPPα), sAPPβ and the MT5-MMP-linked fragment of 95 kDa, sAPP95. However, the lack of MT5-MMP did not affect the activity of β- and γ-secretases. In cultured HEKswe cells, transiently expressed MT5-MMP localized to early endosomes and increased the content of APP and Aβ40 in these organelles, as well as Aβ levels in cell supernatants. This is the first evidence that the pro-amyloidogenic features of MT5-MMP lie, at least in part, on the ability of the proteinase to promote trafficking into one of the amyloidogenic subcellular loci. Together, our data further support the pathogenic role of MT5-MMP in AD and that its inhibition improves the functional and pathological outcomes, in this case in the frontal cortex. These data also support the idea that MT5-MMP could become a novel therapeutic target in AD.

## Introduction

Alzheimer’s disease (AD) is the most common neurodegenerative disorder, characterized by progressive cognitive decline, neuronal/synaptic loss and neuroinflammation. The amyloidogenic hypothesis of the disease postulates that excessive accumulation of amyloid beta peptides (Aβ),—in synergy with tau hyperphosphorylation,—is central to the pathogenic process (De Strooper, 2010; Selkoe and Hardy, 2016). Aβ is produced by the sequential cleavage of amyloid precursor protein (APP) by β-secretase (BACE-1) and by the γ-secretase complex. APP processing produces intermediate APP metabolites such as the β-secretase-derived C-terminal APP fragment of 99 aminoacids (C99), which is also neurotoxic (Lauritzen et al., 2012, 2016). Alternative cleavage of APP by α-secretase (ADAM-10) prevents the formation of Aβ and sets the basis for the non-amyloidogenic pathway (Kuhn et al., 2010). While α-, β- and γ-secretases constitute the classical enzymatic backbone of APP processing, alternative APP processing enzymes are being discovered, including δ- and η-secretases (Andrew et al., 2016). Early work in cultured HEK cells showed that some membrane-type matrix metalloproteinases (MT-MMPs) cleave APP at several sites of the ectodomain (Ahmad et al., 2006). It has been recently shown that the combined cleavage of APP by α-secretase and MT5-MMP (putative η-secretase) at Asn^504^ and Met^505^ (APP_695_ numbering) generates a soluble fragment termed Aη−α, which contains the N-terminal domain of Aβ and displays synaptotoxic properties (Willem et al., 2015). We first reported that APP processing by MT1-MMP and MT5-MMP leads to an increase of Aβ levels in a heterologous cell system (Py et al., 2014; Baranger et al., 2016b), and most important, we demonstrated the contribution of MT5-MMP to AD pathogenesis in the 5xFAD transgenic mouse model of AD (Baranger et al., 2016b). MT5-MMP deficiency in these mice substantially ameliorated their pathological outcome, as illustrated by strong reductions in the neocortex and hippocampus of Aβ and C99 levels, and neuroinflammation, as well as the prevention of deficits in long-term potentiation (LTP) and hippocampal-related spatial learning. Together, these data unveiled MT5-MMP as a new relevant APP processing enzyme with pro-amyloidogenic and synaptotoxic features that contribute to AD pathogenesis.

We sought to extend our early findings on the role of MT5-MMP in the 5xFAD model of the disease and evaluate the impact of MT5-MMP deficiency on frontal cortex dysfunctions characteristic of AD pathology. The frontal cortex controls working memory and executive functions (Stuss and Alexander, 2000; Kane and Engle, 2002; Dalley et al., 2004), which are deeply affected in Alzheimer’s patients at early stages of the disease (Baddeley et al., 1991; Brugger et al., 1996; Perry and Hodges, 1999). Accordingly, we have previously shown that amyloidosis and gliosis correlate with behavior disorders associated to the frontal cortex at early stages of the pathology in 5xFAD mice (Girard et al., 2013). The present study shows that MT5-MMP deficiency protects against pathogenesis in the frontal cortex and prevents learning and memory deficits in bigenic 5xFAD/MT5-MMP^−/−^ mice compared to 5xFAD. Moreover, MT5-MMP deficiency affects APP processing and MT5-MMP expression promotes endosomal trafficking of APP linked to increased amyloidogenesis in a heterologous cell system. Overall, these data reinforce the idea that MT5-MMP plays an important role in AD pathogenesis and further highlight its interest as a potential therapeutic target.

## Materials and Methods

### Mice

The generation of bigenic 5xFAD/MT5-MMP^−/−^ mice (TgMT5^−/−^ thereafter) has been thoroughly described elsewhere (Baranger et al., 2016b). The 5xFAD mouse model of AD (Tg thereafter; Oakley et al., 2006), 5xFAD/MT5-MMP^−/−^, MT5-MMP^−/−^ (MT5^−/−^ thereafter, Komori et al., 2004) and wild-type (WT) control mice, all in C57Bl6 background, were bred in our animal facility. Mice had access to food pellets and water *ad libitum* and were kept under a 12 h light-dark cycle at 21°C. Only 4-month old male mice were used in this study. All experimental procedures were approved by the ethics committee of the Faculty of Medicine (Aix Marseille Université) in accordance with National and European regulations (EU directive N°2010/63), and in agreement with the authorization for animal experimentation attributed to the laboratory by the Prefecture des Bouches-du-Rhône (permit number: D1305508). All efforts were made to minimize animal suffering and to reduce the number of mice used.

### Behavioral Tests

#### Olfactory H-maze (OHM)

**Figure 1:**
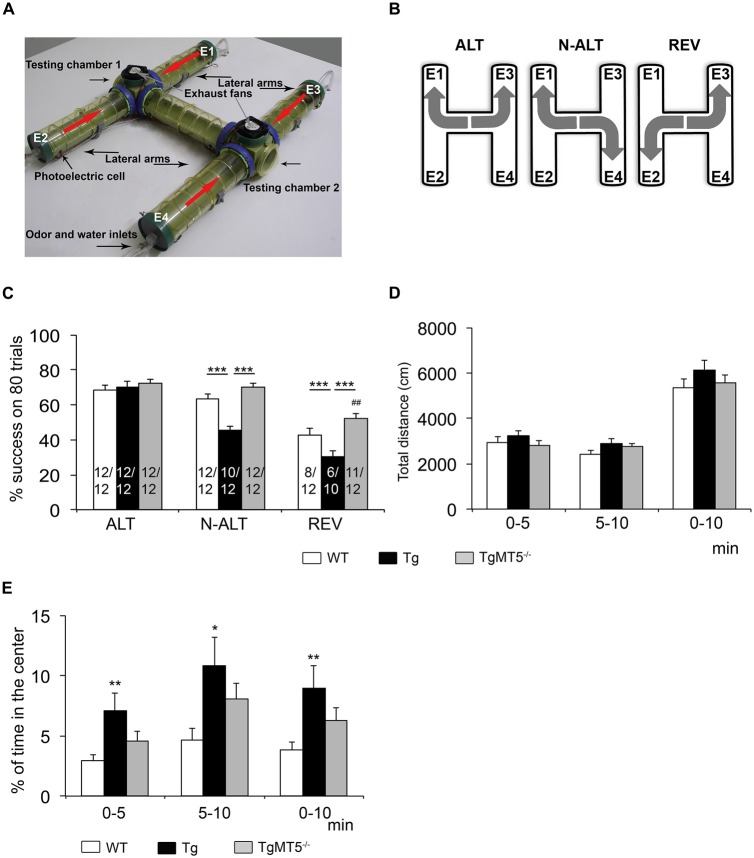
**Cognitive abilities related to frontal cortex are preserved in TgMT5^−/−^ mice. (A)** Photograph showing a top view of the olfactory H-maze (OHM) apparatus. Red arrows indicate the direction of the diffusing odor released by the extremities of the lateral arms E1 and E2 (and alternately E3 and E4) thanks to the inverted exhaust fans placed above each testing chambers. **(B)** Schematic representation of the rule to learn on the alternation (ALT), non-alternation (N-ALT) and reversal (REV) tasks. **(C)** Comparison of the mean percent of success (±SEM) across the 80 trials of each successive tasks (ALT, N-ALT, and REV) for the three groups of mice (WT, Tg and TgMT5^−/−^). The number of successful mice out the total number of mice beginning the task is indicated inside each bar graph. ***P < 0.001 between Tg and either WT or TgMT5^−/−^ and ^##^*P* < 0.01 between TgMT5^−/−^ and WT, ANOVA followed by *post hoc* Fisher’s LSD test. **(D)** Total distance traveled in the open-field test during the two successive blocks of 5 min and during cumulated 10-min session. **(E)** Percentage of time spent by mice in the center of the open field. In **(D,E)**, values are the mean ± SEM of 12 mice per genotype. *P < 0.05, **P < 0.01, ANOVA followed by *post hoc* Fisher’s LSD test.

The olfactory H-maze (OHM) test was performed as previously described (Del’Guidice et al., 2009; Girard et al., 2013). The OHM is an entirely automated behavioral test designed to investigate cognitive functions related to the frontal cortex in mice such as spatial working memory and cognitive plasticity. The OHM is based on the delayed reaction paradigm, which is a specific marker of the frontal cortex function in rodents and humans (Verin et al., 1993). Mice have to discover in the H-shaped maze two different rules consecutively (delayed alternation (ALT) and non-alternation task (N-ALT)) followed by a delayed reversal (REV) task. Mice under water restriction have to learn in which end of the “H” (left or right side of the testing chamber (TC)) they will obtain the reward (a drop of water) according to three consecutive rules (Figures 1A,B).

The OHM is composed of two identical TCs connected by a straight plastic tube. Each TC is connected to two lateral arms, which form altogether an H-shaped tubular structure where mice can move freely (Figure 1A). At the extremity of the lateral arms (E1–E4), water ports allow reward delivery. Each group of two lateral extremities also delivers in an alternating manner a synthetic odor. Thanks to an exhaust fan located above each TC, this odor quickly reaches the TC before being rapidly ejected into the room, which is also equipped with an exhaust ventilation system. The scented air guides mice to the TC where the reward is delivered, but only one side of this TC is associated with the reward. Mice movements are detected by photoelectric cells that generate an infrared beam. When mice interfere with the beam, this triggers water delivery or not, depending on the extremity and the rule.

Odor and water delivery were controlled by a microcomputer running with LabVIEW software (National Instruments France) that also recorded the behavioral data. The experiments were performed in the dark. The experimenter and the computerized recording system were located in an adjacent room and mice observed under infrared camera. The learning procedure was composed of four habituation days followed by one testing day. During the first 4 days mice are habituated to the experimenter and to the maze. Mice placed under water restriction have access to water every habituation day in the extremities of the maze lateral arms in the presence of the odor. The testing session consists in discovering the three consecutive tasks: ALT, N-ALT and REV. From a previous study (Belhaoues et al., 2005), the success criterion was set at four consecutive correct trials and then the rule switched automatically. The session ended when the rules of three successive tasks were discovered. Mice that did not succeed within the limits of either 1 h-trial or 80 trials were excluded from the study and did not continue on the following tasks. Mice performance was compared using the mean percentage of success, which was obtained with the sum of cumulated percentage of success divided by eighty, the maximum number of trials for each task (see for details, Girard et al., 2013).

#### Open Field Test

The open field test was used to assess spontaneous locomotor activity and exploratory behavior in the same mice that performed the OHM. The open field was a white plastic square of 50 cm on each side and walls 45 cm high. Testing was carried out in a dimly illuminated room (40 lux) and recorded by an automated tracking system using a video camera mounted above the apparatus (Viewpoint VideoTrack version 3.0, Champagne Au Mont D’or, France). Animals were placed in the center of the field and the traveled distance measured in one 10-min session. In order to identify a possible heterogeneity in mice behavior across time, we analyzed the average scores in each of the 5-min intervals as well as the average of the entire 10-min session. Moreover, we measured the percentage of time spent by mice in the center of the arena (20 cm × 20 cm) with respect to the total time following the same time intervals as above. The square was cleaned at the end of each session.

### Immunohistochemistry, Image Analysis and Quantifications

Mice were deeply anesthetized with an intraperitoneal injection of 355 mg/kg sodium pentobarbital (Ceva Santé Animal, Libourne, France) and transcardially perfused with cold saline solution (NaCl 0.9%), followed by fixation with Antigenfix solution (Diapath, MM France, Brignais, France). Brains were then post-fixed 24 h in the same fixative and stored (4°C) in phosphate buffer saline (PBS), pH 7.4. Free-floating coronal sections (30 μm thick) were obtained using a vibratome (Microm HM 650V, Thermo Scientific, MA, USA) and stored at −20°C in a cryoprotectant solution (30% glycerol, 30% ethylene glycol, 30% ultra-pure water and 10% of 0.2M PBS). Sections were pre-incubated in a blocking solution (PBS, 3% BSA and 0.1% Triton X-100), followed by overnight incubation at 4°C with anti-GFAP (1/300, Dako France, Trappes, France), anti-Iba1 (1/300, Wako, Sobioda, Montbonnot-Saint-Martin, France), anti-Aβ 6E10 (1/300, Ozyme, Saint Quentin en Yvelines, France), and the corresponding secondary anti-rabbit or anti-mouse antibodies coupled to Alexa Fluor^®^ 488 and 568 (Life Technologies, Saint Aubin, France) for 3 h at room temperature. Nuclei were stained with 0.5 μg/mL DNA intercalant Hoechst #33258 (Life Technologies). Sections were mounted using Prolong Gold Antifade reagent (Life Technologies). Negative control immunostaining was performed without the primary antibody, and no immunoreactivity was confirmed. Samples were observed under a LSM 700 confocal or Axiovert inverted microscope (Zeiss, Jena, Germany). Images were analyzed using the Axiovision (Zeiss), Photoshop (Adobe) and ImageJ (NIH) softwares.

The number of plaques was blindly scored in two brain sections per animal by three investigators at bregma 2.68 and 2.46 and normalized by the surface of the brain region analyzed. The average area of individual plaques was measured using the analyze particles plugin from Image J. “Dense core plaques” were identified as having a compact spherical appearance without scattered amyloid granules/fibrillar-like structures around, whereas “diffuse plaques” exhibited a halo of granular/fibrillar-like structures around a center area where a compact amyloid core was or was not present*.* Images of immunostaining for glial markers were acquired using identical exposure times and settings across the different groups. Background was substracted from these images using the Image J rolling ball radius algorithm. The same fluorescence threshold and binarization settings were applied to all images before measuring the immunostained area over the total area.

### Western Blot Biochemical Analysis

The detection of APP fragments in different fractions was carried as previously described with, slight modifications (Baranger et al., 2016b). To detect the soluble forms sAPPα and sAPPβ, male mice were deeply anesthetized as indicated above and transcardially perfused with cold saline solution (NaCl 0.9%). The frontal cortex was microdissected and snap-frozen with cold isopentane for biochemical assays. To obtain insoluble and soluble dyethylamine (DEA)-extracted fractions, frozen tissue was homogenized in 500 μL of cold DEA buffer (50 mM NaCl, 2 mM EDTA, 0.2% DEA, proteinase inhibitor cocktail (Millipore) with a rotor-stator homogenizer, directly followed by neutralization of the homogenate with Tris-HCl buffer, pH 6.7. Homogenate was centrifuged at 5000 r.p.m. for 5 min. Supernatants were transferred to new vials and subjected to clarifying ultracentrifugation at 55,000 r.p.m. for 30 min using a TLA55 rotor. Pellets were washed with PBS to remove the remaining soluble fraction and then lysed in STE buffer (150 mM NaCl, 50 mM Tris-HCl, 2 mM EDTA) and 1% Triton for 60 min on ice followed by centrifugation at 14,000 r.p.m for 10 min at 4°C. The resulting insoluble fractions were transferred to fresh tubes. Protein concentration of insoluble or DEA fractions was measured using the Bradford (BCA) method according to the manufacturer’s instructions (Interchim, Mannheim, Germany). Proteins were separated on 10% SDS-PAGE gels, and then transferred to a 0.45 mm nitrocellulose membrane (Whatman^®^, Maidstone, Kent, UK). After blocking, membranes were probed with the following primary antibodies: anti-APP N-ter, clone 22C11 (1/1000, Millipore), anti-APP C-Ter, clone 2C11; anti-mAPPs-α, clone 7A6; anti-hAPPs-α, clone 14D6; anti-APPs-β wt, clone 192 wt and anti-hAPPs-β swe, clone 192 swe (both kindly provided by Dale Schenk) and anti-β-actin (Sigma-Aldrich, Saint Quentin-Fallavier, France). Blots were developed using appropriate horseradish peroxidase-conjugated secondary IgG antibodies (Promega Corp., Madison, WI, USA and anti-rat HRP, Santa Cruz Biotechnology Inc., Dallas, TX, USA) and the ECL chemiluminescence system (GE Healthcare). Quantification of western blots was performed using Fuji Las-4000 software (Fuji Film inc., Minato, Tokyo, Japan) and Image J.

For the analysis of 6E10^+^ and N-cadherin fragments, GFAP, PSD-95, synaptophysin and β-III tubulin, male mice were anesthetized and brains dissected as indicated above. Samples were homogenized in 25% w/v of 50 mM Tris-HCl, pH 7.5 buffer containing 150 mM NaCl, 2 mM EDTA, 1% Triton X-100, 0.05% SDS and proteinase inhibitor cocktail (Millipore) and centrifuged at 10000× *g* for 10 min at 4°C. This “soluble fraction” contained cytosolic proteins and proteins easy to solubilize. Cell pellets were resuspended in 25% w/v of 50 mM Tris-HCl pH 7.5 buffer containing 2% SDS, then sonicated and centrifuged at 10000× *g* for 10 min at 4°C to obtain an “insoluble fraction” containing more insoluble proteins, among which an important part of membrane proteins (Reinés et al., 2012). Protein concentrations were determined using a Bio-Rad *DC*^TM^ protein assay kit (Bio-Rad, Marnes-La-Coquette, France) and 50 μg of protein were run on 10%–15% SDS-PAGE gels and transferred to nitrocellulose membranes (Amersham Bioscience, Velizy-Villacoublay, France; Py et al., 2014). After blocking, membranes were probed with the following antibodies: anti-GFAP (1/1000, Millipore), anti-Aβ 6E10 (1/300, Ozyme), anti-APP C-terminal fragment (APP-CTF, 1/2000, Sigma-Aldrich), anti-N-cadherin CTF (1/1000, BD Biosciences, Le Pont de Claix, France), anti-PSD-95 (1/2000, Millipore), anti-synaptophysin (1/2000, Millipore), anti-β-III tubulin (1/3000, Sigma-Aldrich), anti-GAPDH (1/5000, Millipore) and anti-β-actin (1/5000, Sigma-Aldrich), and then incubated with appropriate horseradish peroxidase-conjugated secondary IgG antibodies (Jackson Immunoresearch, West Grove, PA, USA). Immunoblot signals were visualized using the ECL chemiluminescence kit (GE Healthcare, Dutscher, Brumath, France) and quantified using ImageJ software.

For the detection of APP CTF CTFα and CTFβ, respectively derived from APP processing by α- and β-secretases, PS1 and nicastrin, solubilized membranes were used as reported previously (Sevalle et al., 2009; Baranger et al., 2016b). Briefly, frontal cortex samples were resuspended in 10 mM Tris–HCl buffer, pH 7.5 with proteinase inhibitors (Sigma-Aldrich), subjected to repeated passages through a 25G needle and first centrifuged at 800× *g* for 10 min at 4°C. The resulting supernatants were centrifuged at 20,000× *g* centrifugation for 1 h at 4°C. Pellets containing the membranes were then resuspended in solubilization buffer, 150 mM sodium citrate, pH 6.4 with 3-[(3-cholamidopropyl) dimethylammonio]-2-hydroxy-1-propanesulfonate (CHAPSO, 1% (v/v)) and proteinase inhibitors (Millipore). Samples were diluted in solubilization buffer at (1 mg/mL). The denatured samples were analyzed by western blot using APP-CTF (1/2000, Sigma-Aldrich), anti-PS1 N-terminal (1/1000, provided by Paul Fraser), and anti-nicastrin (1/1000, Sigma-Aldrich) antibodies, and normalized using an anti-tubulin antibody (1/5000, Sigma-Aldrich).

### BACE-1 Activity Assay

To assess β-secretase activity, 15 μg of proteins from solubilized membranes were used as previously described (Andrau et al., 2003). Briefly, samples were incubated in a final volume of acetate buffer (25 mM, pH 4.5, 100 μL) containing 10 μM of BACE-1 substrate (7-methoxycoumarin-4-yl)-acetyl-SEVNLDAEFRK(2, 4-dinitrophenyl)-RRNH_2_ (R&D Systems, Bio-Techne, Lille, France) with or without β-secretase inhibitor I (75 μM; PromoCell, Heidelberg, Germany). BACE-1 activity corresponds to the β-secretase inhibitor I-sensitive fluorescence recorded at 320 (excitation) and 420 (emission) nm wavelengths, using a FLUOstar Omega spectrofluorometer (BMG Labtech, Palaiseau, France).

### Plasmid Construction

The MT5-MMP cDNA was amplified by PCR from C57Bl6 mouse cerebellum, and cloned as described earlier for other MMPs (Sbai et al., 2008; Ould-Yahoui et al., 2009; Sbai et al., 2010). We used the following primers: *MT5For* ATA TAT GAA TTC GGA TGC CGA GGA GCC GGG GAG GCC GCG CTG and *MT5Rev* ATA TAT GTC GAC AGT ACC CAC TCC TGG ACC GGC CGC TTA TAG TAG and cloned into pEGFP-N1 (Clontech, Saint-Germain-en-Laye, France). This cDNA construct is referred to as MT5. Green fluorescent protein (GFP) and MT5 plasmids were amplified in DH5α* E. coli* (Life Technologies) and purified using the NucleoBond Xtra Midi Plus EF (Macherey-Nagel, Hoerd, France) according to the manufacturer’s recommendations.

### Transferrin Pulse Chase and Colocalization of APP/Aβ in the Endosomal System

The impact of MT5-MMP on APP intracellular trafficking was studied on HEKswe cells stably transfected with pcDNA3 coding for human APP harboring the Swedish mutation (Marambaud et al., 1998). To perform immunocytochemistry, 6.10^4^ cells/well were plated on coverslips for 24 h and then transfected with a plasmid coding for MT5-MMP (MT5) fused to GFP in the C-terminal end. GFP plasmids were used as controls (Baranger et al., 2016b). Endosomes were labeled with 0.25 mg/mL Alexa Fluor^®^ 647-transferrin (Tf; Life Technologies) for 30 min, which mostly labels early endosomes in HEK cells (Kaether et al., 2006a). Cells were then fixed for 15 min at room temperature with Antigenfix solution (Diapath) and incubated for 1 h at room temperature in a PBS blocking solution containing 0.1% Triton X-100 and 3% BSA. Cells were then incubated overnight at 4°C with anti-Aβ 6E10 (1/300, Ozyme) and anti-GFP (Millipore) antibodies. Endosomes were labeled using antibodies against anti-EEA1 (Early Endosome Antigen 1, 1/300, Santa-Cruz Biotechnology, Heidelberg, Germany), anti-Rab11 (1/300, BD Biosciences) and anti-Rab7 (1/300, Abcam, France) to respectively label early, recycling and late endosomes/lysosomes. Anti-Aβ40 antibody (Ozyme) was used to specifically recognize the genuine Aβ40 C-terminal end generated after γ-secretase cleavage. The coverslips were rinsed in PBS 1X and then incubated with the appropriate Alexa Fluor^®^-conjugated secondary antibodies (Life Technologies) for 1 h at room temperature. Nuclei were stained with Hoechst #33258 for 10 min (0.5 μg/mL, Life Technologies). Omission of the primary antibody was used as negative control and no immunostaining was observed. Coverslips were mounted using Prolong Gold Antifading reagent on Superfrost glass slides (Life Technologies).

Images were taken and processed using a confocal microscope (LSM 700) and Zen software (Zeiss). Between five and seven stacks of 0.4 μm per cell were generated and colocalization measured in each single stack using the Jacop plugin of ImageJ (Bolte and Cordelieres, 2006). We analyzed at least 20 cells per group and per independent experiment. To analyze the impact of the proteins expressed following transfection on APP/Aβ content, we quantified the percentage of colocalization between Aβ 6E10 and Alexa Fluor^®^ 647-Tf, and colocalization between Aβ40 and, EEA1, Rab11 and Rab7. We also performed a qualitative analysis of MT5-MMP/GFP distribution in endosomal vesicles labeled by Alexa Fluor^®^ 647-Tf. Finally, we evaluated possible changes in the content of endosomes by measuring the cell area occupied by Alexa Fluor^®^ 647-Tf in each experimental condition relative to the total cell area.

### ELISA Assays

For detection of Aβ38, Aβ40 and Aβ42 in the DEA fractions from the frontal cortex, we used the MSD Aβ Triplex sandwich immunoassay (MesoScale Discovery, Rockville, MD, USA). MSD C-terminal-specific antibodies were pre-spotted into each well. For detection, a ruthenylated 6E10 antibody was used. The concentrations of human Aβ peptides were normalized to protein content and calculated using the MSD Discovery Workbench software. For detection of Aβ40 levels in cell supernatants of HEKswe cells, the human Aβ40 ELISA kit (Life Technologies) was used according to the manufacturer’s recommendations. The γ-secretase inhibitor DAPT (Tocris, Bio-Techne, Lille, France), 10 μM for 48 h, was used to inhibit Aβ production. The levels of proinflammatory mediators interleukin-1β (IL-1β), tumor necrosis factor alpha (TNF-α) and monocyte chemoattractant protein-1 (MCP-1) in triton-soluble fractions were determined using the murine ELISA Development Kit (Peprotech, Neuilly-sur-Seine, France) according to the manufacturer’s recommendations.

### Statistics

Significant differences between groups were determined by a one-way ANOVA followed by *post hoc* Fisher’s LSD for multiple comparisons. Two-tailed unpaired Student *t*-tests were used to compare two experimental groups. The analysis of OHM data was performed using repeated-measures (MANOVA) across the entire session and then by one-way ANOVA for each task followed by *post hoc* Fisher’s LSD for multiple comparisons. In all cases we used the Kaleida Graph software. Values represent the means ± SEM of the indicated number of independent experiments/animals, and the level of significance was set at *P* < 0.05.

## Results

### MT5-MMP Deficiency in 5xFAD Mice Prevents Learning Deficits in the Olfactory H-maze Task without Alterations of Locomotion

The comparison of the mean percentage of success on 80 trials across the three groups (WT, Tg and TgMT5^−/−^) of 4-month old male mice (Figure 1C) revealed a substantial difference between Tg and the WT and TgMT5^−/−^ mice in their ability to perform the three successive tasks (MANOVA Task × Group interaction: *F*_(2,237)_ = 32.97; *P* < 0.001), with a significant group difference (MANOVA: *F*_(2,237)_ = 8.35; *P* < 0.001). All the groups scored a similar mean percentage of success in the first task (ALT; ANOVA: *F*_(2,237)_ = 0.35; ns), but a significant difference was observed in the N-ALT and REV tasks (ANOVAs: *F*_(2,237)_ ≥ 19.39; *P* < 0.001). Indeed, analysis of the N-ALT and REV tasks revealed that Tg mice displayed significantly lower levels of performance on these two tasks compared to WT and TgMT5^−/−^ (Figure 1C). In addition, a significant difference was also observed between WT and TgMT5^−/−^ on the last task (REV; Figure 1C). It has to be noted that on the N-ALT task, two Tg mice failed to solve the task, while all the WT and TgMT5^−/−^ were successful. On the REV task, four more Tg mice and four WT failed to meet the criterion, while only one TgMT5^−/−^ did not reach the criterion. All the mice were submitted to the open-field paradigm (Figure 1D). The three groups of mice showed similar locomotor activity during the 10-min session (ANOVAs: *F*_(2,33)_ ≤ 1.84; ns; Figure 1D), indicating that the differences observed in the OHM were not the result of altered locomotor activity. However, Tg mice spent more time in the center of the arena than WT mice (ANOVAs: *F*_(2,33)_ ≥ 3.29; *P* < 0.05), suggesting that Tg mice were less anxious. TgMT5^−/−^ mice scores were between WT and Tg values and showed no statistical differences with respect to either group (Figure 1E).

### Decreased Aβ Plaques and Neuroinflammatory Response in the Frontal Cortex Of TgMT5^−/−^ Mice

**Figure 2:**
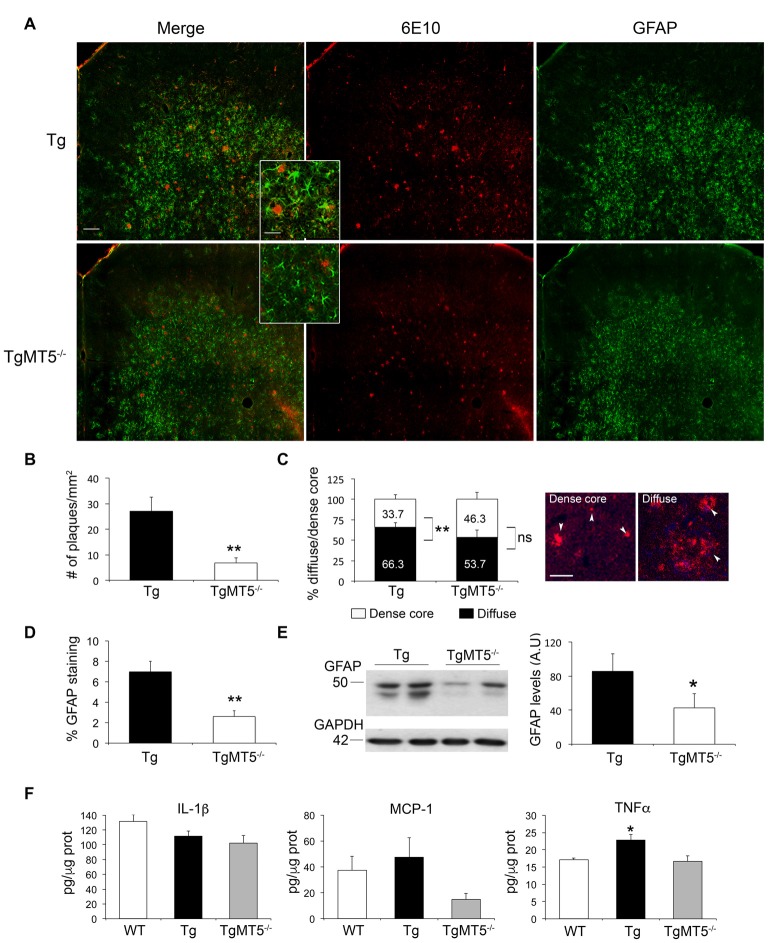
**Decreased amyloid burden and neuroinflammation in the frontal cortex of TgMT5^−/−^ mice compared to Tg. (A)** Representative epifluorescence microphotographs showing a strong decrease of 6E10 and GFAP immunostaining in coronal sections of frontal cortex from TgMT5^−/−^, compared Tg mice; scale bar 100 μm. Insets represent high power magnification from selected fields; scale bar 20 μm. **(B)** Quantification of the number of plaques over the area of frontal cortex in Tg and TgMT5^−/−^ mice. Values are the mean ± SEM of 5–6 brains per genotype. **P < 0.01; Student’s *t* test. **(C)** Quantification of the percentage of dense core and diffuse plaques over the total measured area of frontal cortex in Tg and TgMT5^−/−^ mice. On the right, representative epifluorescence microphotographs of both plaque phenotypes stained with the 6E10 antibody (arrowheads). Values are the mean ± SEM of 5–6 brains per genotype. **P < 0.01; Student’s *t* test; ns, non significant. **(D)** Quantification of changes in GFAP immunostaining as the percentage of the immunostained area/total area in the frontal cortex. Values are the mean ± SEM of 5–6 brains per genotype. **P < 0.01, Student’s *t* test. **(E)** Representative western blot (left) and the corresponding quantification (right) in arbitrary units (A.U.), showing GFAP immunoreactivity in the soluble fraction of frontal cortices from Tg and TgMT5^−/−^ mice. Values are the mean ± SEM of 5–6 brains per genotype. *P < 0.05, Student’s *t* test. **(F)** ELISA assay showing quantification of IL-1β, MCP-1 and TNF-α levels (pg/μg of protein) in the soluble fraction of frontal cortices from WT, Tg and TgMT5^−/−^ mice. Values are the mean ± SEM of 5–6 mice per genotype. *P < 0.05, ANOVA followed by *post hoc* Fisher’s LSD test.

In agreement with our previous description of the 5xFAD model (Girard et al., 2013), amyloid plaques were abundant in the frontal cortex at 4 months of age. This is illustrated in Figure 2 by immunohistochemistry with the 6E10 antibody, which recognizes the N-terminal domain of the human Aβ sequence. Compared to Tg, TgMT5^−/−^ mice exhibited a 75% significant decrease in the number of plaques (Figures 2A,B), which were smaller in average (323 ± 57 μm^2^) compared to Tg (519 ± 122 μm^2^), although it did not reach statistical significance. Moreover, 66.3% of plaques in Tg brains had a diffuse phenotype with or without a dense core inside, while only the remaining 33.7% presented an amyloid dense core without surrounding deposits. These statistically significant differences found in Tg mice disappeared in the frontal cortex of TgMT5^−/−^ mice, with a 53.7%/46.3% diffuse vs. dense ratio (Figure 2C).

Reduction of amyloid burden in the frontal cortex of TgMT5^−/−^ mice was concomitant with a strong 81% reduction in astroglial reactivity, as demonstrated by quantification of GFAP immunostaining (Figure 2D), further confirmed by a 50% decrease in GFAP levels measured by western blot (Figure 2E). No significant differences were observed in microglial reactivity evaluated with an Iba1 antibody (not shown). Seemingly the levels of two pro-inflammatory mediators commonly associated with Alzheimer’s pathology, IL-1β and MCP-1 remained stable between WT, Tg and TgMT5^−/−^ mice, although a tendency to decrease in TgMT5^−/−^ tissue was observed (Figure 2F). In contrast with IL-1β and MCP-1, TNF-α levels increased by 33% in Tg with respect to WT mice, and most interestingly, such increase was not detected in TgMT5^−/−^ mice (Figure 2F).

### Unchanged Levels of Markers of Neuronal and Synaptic Integrity

**Figure 3:**
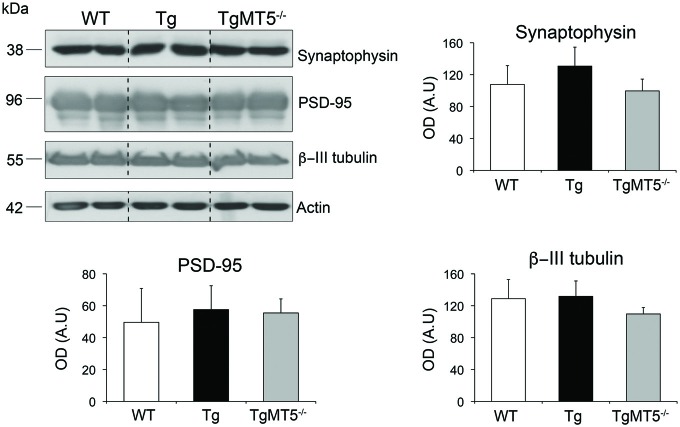
**The levels of synaptic and neuronal markers are not affected in the frontal cortex of TgMT5^−/−^ mice.** Representative western blots for synaptophysin and β-III tubulin (soluble fraction) and PSD-95 (insoluble fraction) of frontal cortices from WT, Tg and TgMT5^−/−^ mice. The actin-normalized values show no differences between the three genotypes. Values are the mean ± SEM of 5–6 mice per genotype. ANOVA followed by *post hoc* Fisher’s LSD test. Vertical dashed lines on western blots separate non-contiguous bands from the same gel.

Dysfunctional synaptic plasticity correlates well with learning deficits in AD (Selkoe, 2008). In addition, it has been suggested that MT5-MMP could work in synergy with γ-secretase to disrupt synaptic integrity and hence synaptic transmission (Restituito et al., 2011). Accordingly, we examined whether the integrity of synapses or the neuronal network could be compromised in the frontal cortex of Tg mice with impaired learning, and improved by the lack of MT5-MMP. The pre-synaptic marker synaptophysin and neuronal specific marker β-III tubulin were equally distributed across WT, Tg or TgMT5^−/−^ genotypes in the triton-soluble (Figure 3) or insoluble (not shown) fractions. The post-synaptic marker PSD-95 was mainly detected in the insoluble fraction (Figure 3) and its levels remained also stable across genotypes, indicating altogether that the structure of the neuronal network was relatively well preserved at the 4-month stage of the pathology.

### Decreased Levels of Soluble Aβ, and Various N-terminal and C-terminal APP Fragments in the Frontal Cortex of TgMT5^−/−^ Mice

**Figure 4:**
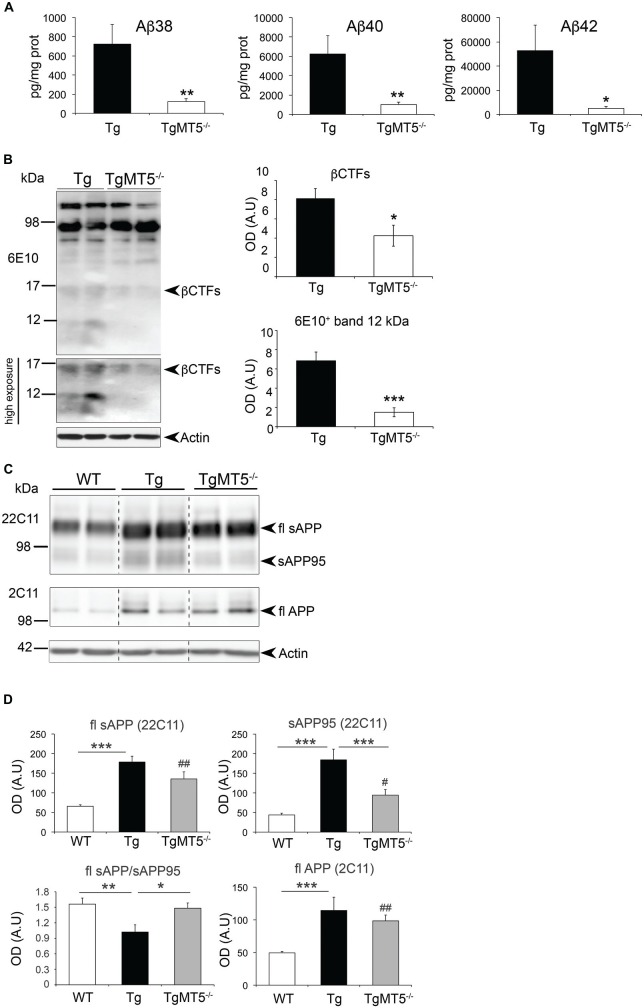
**Amyloid precursor protein (APP) metabolism is reduced in the frontal cortex of TgMT5^−/−^ mice compared to Tg.** **(A)** ELISA quantification (pg/mg protein) of Aβ38, Aβ40 and Aβ42 levels in DEA fractions. Values are the mean ± SEM of 5–6 mice per genotype. *P < 0.05, **P < 0.01, Student’s *t* test. **(B)** Representative western blots (left) showing 6E10^+^ immunoreactivity in the insoluble fraction of the frontal cortex. The lower western blot has been highly exposed to highlight the decrease of βCTFs and the ~12 kDa band in TgMT5^−/−^ compared to Tg mice, and the appearance of diffuse immunoreactivity in Tg samples below around 6 kDa. The plots (right) show the corresponding quantifications for the βCTFs and the ~12 kDa band. Values are the mean ± SEM of 5–6 actin-normalized optical densities (OD); A.U., arbitrary units. *P < 0.05, ***P < 0.001, Student’s *t* test. **(C)** Western blot showing full length (fl) APP in the soluble (22C11 antibody) and insoluble (2C11 antibody) fractions. Note that the levels of a soluble 95 kDa short form of APP (sAPP95) are downregulated in TgMT5^−/−^ brains compared to Tg. **(D)** The corresponding quantification of APP levels in the soluble fraction of frontal cortex from WT, Tg and TgMT5^−/−^ mice, using the 22C11 (N-ter) and 2C11 (C-ter) antibodies. Actin-normalized values are the mean ± SEM of 5–6 mice per genotype. *P < 0.05, **P < 0.01, ***P < 0.001 between Tg and either WT or TgMT5^−/−^; ^#^*P* < 0.05, ^##^*P* < 0.01 between TgMT5^−/−^ and WT, ANOVA followed by *post hoc* Fisher’s LSD test. Vertical dashed lines on western blots separate non-contiguous bands from the same gel.

The striking decrease in the content of amyloid plaques upon MT5-MMP deficiency paralleled similar reductions in the levels of soluble Aβ species, including Aβ38 (−83%), Aβ40 (−84%) and Aβ42 (−90%; Figure 4A). We also detected significant reductions of ~12 and ~15 kDa 6E10^+^ bands in frontal cortex homogenates of TgMT5^−/−^ mice compared to Tg (Figure 4B). The sizes of these bands are compatible with the expected size for Aβ trimers or Aη−α and βCTF C99, respectively.

Further changes in APP metabolism were detected when analyzing the levels of soluble APP (sAPP) in DEA soluble fractions (Figures 4C,D); the 22C11 antibody, which recognizes the N-terminal domain of APP, revealed a ~110 kDa band that was respectively upregulated in Tg and TgMT5^−/−^ brains by 2.73- and 2.05-fold, compared to WT, while no differences were observed between Tg and TgMT5^−/−^ (Figures 4C,D). A lower band of ~95 kDa (sAPP95) was also significantly upregulated by 4.2-fold in Tg frontal cortex with respect to WT. The sAPP95 fragment was not detected with the 6E10 or APP-CTF antibodies (not shown). However, sAPP95 levels dropped by 49% in TgMT5^−/−^ frontal cortices with respect to Tg, but were still 2.1-fold above WT values (Figures 4C,D). Consequently, the sAPP/sAPP95 ratio in WT and TgMT5^−/−^ tissues was 1.5-fold higher than in Tg (Figure 4D). APP levels in the insoluble fraction were measured using the APP C-terminal antibody 2C11, which detected a ~130 kDa band likely representing the canonical protein (full-length, fl; Figure 4C). Consistent with the presence of an additional copy of human *APP* in transgenic mice, higher levels of flAPP were detected in the frontal cortex of Tg (2.3-fold) and TgMT5^−/−^ (2-fold) mice compared to WT, and no significant differences were observed between Tg and TgMT5^−/−^ (Figure 4D).

**Figure 5:**
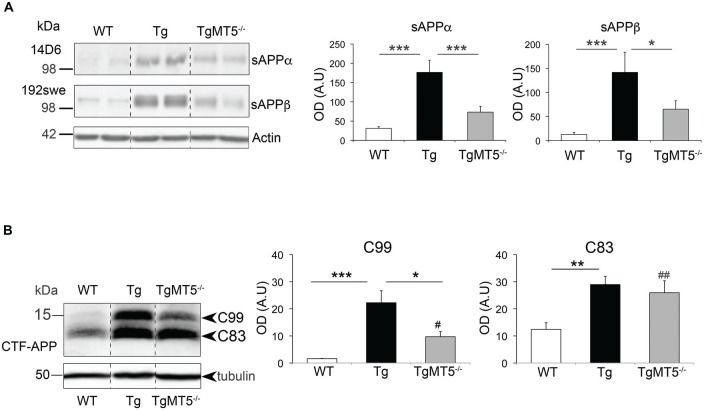
**sAPPα, sAPPβ and C99 levels are strongly decreased in the frontal cortex of TgMT5^−/−^ mice compared to Tg. (A)** Western blot analysis (left) and quantification (right) of APP levels using specific antibodies against human sAPPα (14D6) and sAPPβ (192swe) that recognize the neoepitopes generated after α- or β-secretase cleavage. Note that the increase observed in Tg mice was prevented by MT5-MMP deficiency in TgMT5^−/−^ mice. Actin-normalized values are the mean ± SEM of 5–6 mice per genotype. *P < 0.05, ***P < 0.001, ANOVA followed by *post hoc* Fisher’s LSD test. OD, optical density; A.U., arbitrary units. **(B)** Western blot analysis (left) and quantification (right) of APP C-terminal fragment (CTF) C99 and C83 in solubilized membranes using the CTF-APP antibody, which recognizes the C-terminal end of APP. Tubulin-normalized values are the mean ± SEM of 5–6 mice per genotype. OD, optical density; A.U., arbitrary units *P < 0.05, **P < 0.01, ***P < 0.001 between Tg and either WT or TgMT5^−/−^ and ^#^*P* < 0.05, ^##^*P* < 0.01 between TgMT5^−/−^ and WT, ANOVA followed by *post hoc* Fisher’s LSD test. Vertical dashed lines on western blots separate non-contiguous bands from the same gel.

The data above suggested that MT5-MMP deficiency could affect APP processing N-terminally and therefore impact on the generation of sAPPα and sAPPβ, the soluble N-terminal fragments released by α- and β-secretase, respectively. Thus sAPPα and sAPPβ levels can be considered as an indirect readout of the activity of these secretases (Kuhn et al., 2010). Accordingly, we used the 14D6 and 192swe antibodies, which specifically recognize the C-terminal neoepitopes of sAPPα and sAPPβ (Figure 5A). The levels of sAPPα and sAPPβ respectively augmented by 5.7- and 11.2-fold in Tg compared to WT. Such increases were strongly attenuated in the frontal cortex of TgMT5^−/−^ mice, whose levels significantly dropped 58.5% (sAPPα) and 54% (sAPPβ) compared to Tg, and nearly reached statistical significance with respect to WT values (*P* = 0.058 for sAPPα; *P* = 0.057 for sAPPβ; Figure 5A).

We used an APP-CTF antibody to assess the impact of the genotype on the levels of the remaining membrane bound C-terminal APP fragments, after cleavage by α- and β-secretase cleavage (Figure 5B). Thus, we detected in WT samples a doublet of ~15 kDa representing C83 and much less abundant C99. Both C83 and C99 were largely upregulated in Tg brains, 2.4-fold for C83 and 9.1-fold for C99. Most important, C99 levels were strongly reduced by 61.7% in TgMT5^−/−^, compared to Tg mice, but were still significantly higher (4-fold) than WT levels. In contrast, C83 levels were not reduced in TgMT5^−/−^ compared to Tg samples and remained significantly upregulated by 2-fold with respect to WT (Figure 5B).

### Preserved β- and γ-secretase Activity in the Frontal Cortex of TgMT5^−/−^ Mice

**Figure 6:**
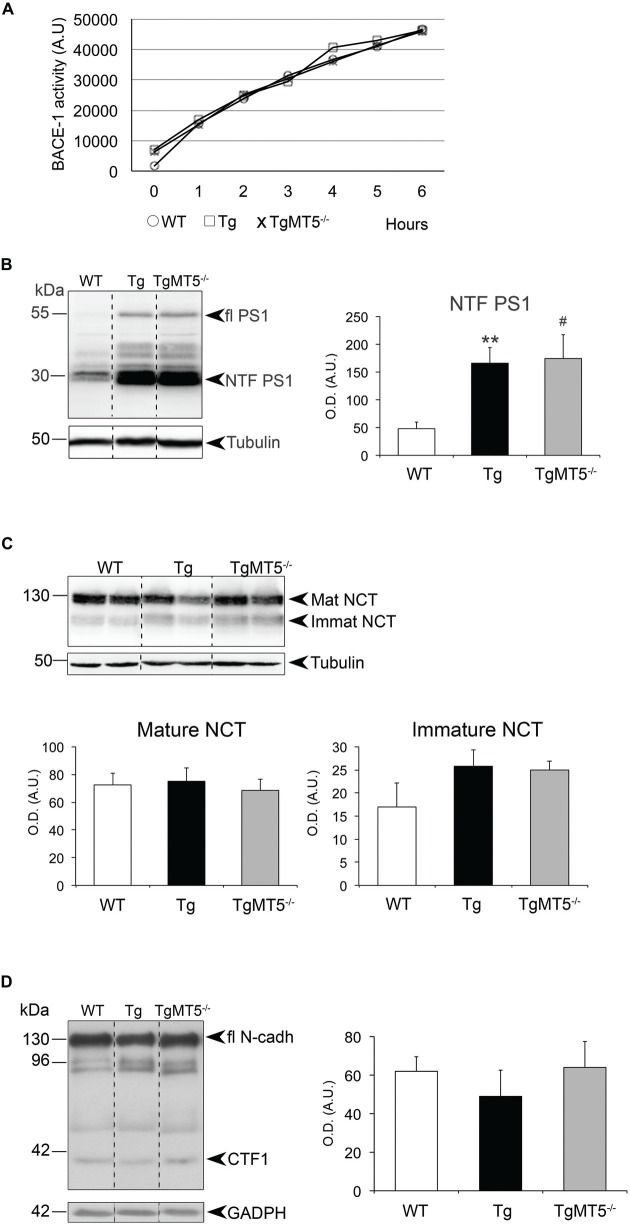
**β- and γ-secretase activities are not altered in the frontal cortex of TgMT5^−/−^ mice compared to Tg. (A)** Plots showing no differences of β-secretase activity in solubilized membranes from WT, Tg and TgMT5^−/−^, using a specific fluorogenic substrate. **(B)** Western blot analysis (left) and quantification (right) of the N-terminal fragment of PS1 (PS1-NTF), resulting from PS1 autocatalysis, in solubilized membranes of the frontal cortex. Tubulin-normalized values are the mean ± SEM of 5–6 mice per genotype. **P < 0.01, between Tg and WT and ^#^*P* < 0.05, between WT and TgMT5^−/−^, ANOVA followed by *post hoc* Fisher’s LSD test. **(C)** Western blot analysis (upper) and quantification (lower) of mature and immature nicastrin levels in solubilized membranes showing no differences across groups. Tubulin-normalized values are the mean ± SEM of 5–6 mice per genotype. **(D)** Western blot analysis (left) and quantification (right) of N-cadherin CTF levels in insoluble fractions showing no differences across groups. GAPDH-normalized values are the mean ± SEM of 5–6 mice per genotype. OD, optical density; A.U., arbitrary units. Vertical dashed lines on western blots separate non-contiguous bands from the same gel.

The observed reduction of sAPPβ levels in TgMT5^−/−^ could reflect a deficit of β-secretase activity, which could ultimately contribute to the anti-amyloidogenic effects of MT5-MMP deficiency. We further investigated this contingency using an enzymatic assay for BACE-1 activity (Andrau et al., 2003), the main β-secretase that catalyzes the rate-limiting step for Aβ production. The *in vitro* test on homogenates from WT, Tg and TgMT5^−/−^ frontal cortices did not detect any genotype-associated alteration of BACE-1 processing a specific fluorogenic substrate over a 6 h-time period (Figure 6A).

To determine whether changes in the content of C99 were related with alterations of γ-secretase activity *per se*, we leveraged on the autocatalytic activity of PS1, the main proteolytic unit of the γ-secretase complex. We detected by western blot the ~55 kDa full-length form and a ~30 kDa N-terminal fragment (NTF-PS1) resulting from PS1 autocatalysis (Figure 6B). As anticipated, transgenic mice carrying an additional copy of the mutated human *PSEN1* gene exhibited higher levels of PS1 compared to WT, but there were no differences in processed NTF-PS1 content, indicating similar γ-secretase-mediated endoproteolysis activity in Tg and TgMT5^−/−^ mice (Figure 6B). Seemingly, there was no difference between genotypes in the content of nicastrin, another member of the γ-secretase complex necessary for substrate recognition. This is illustrated in Figure 6C, which shows no changes in the immature and mature forms of nicastrin, respectively associated with pre-assembled and assembled γ-secretase complex, suggesting altogether that the enzymatic complex is intact (Figure 6C). The finding that another γ-secretase substrate, the 37 kDa C-terminal domain of N-cadherin, was similarly processed across genotypes, further supported a genotype-independent catalytic activity of γ-secretase (Figure 6D).

### MT5-MMP Modulates the Content of APP-derived Species in the Endosomal System

**Figure 7:**
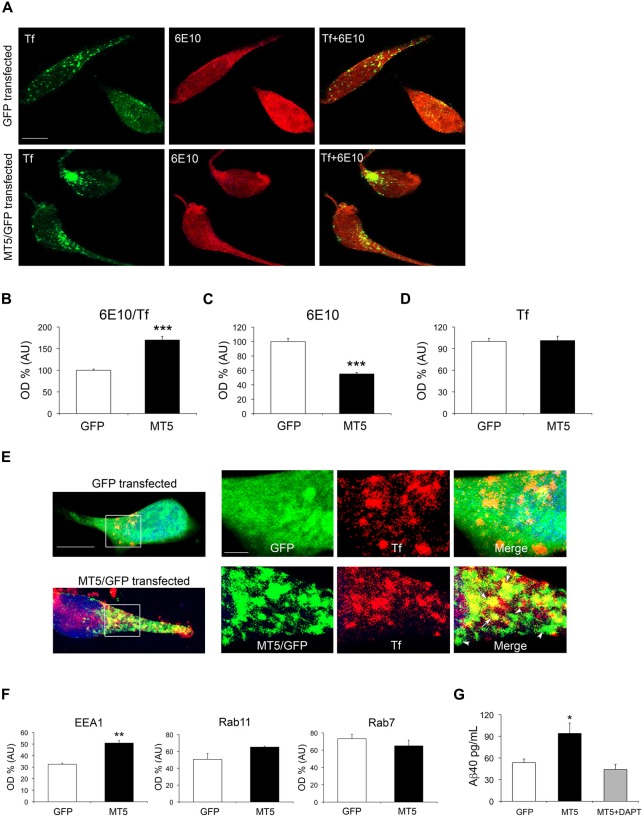
**MT5-MMP promotes APP/Aβ distribution in early endosomes and increases Aβ levels in the supernatant of HEKswe cells. (A)** Representative epifluorescence microphotographs of HEKswe cells after transfection with green fluorescent protein (GFP) and MT5-MMP/GFP (MT5/GFP) plasmids, showing from left to right: transferrin (Tf, green) labeling representing early endosomes, 6E10 immunostaining (red), the merge of both (Tf+6E10), where yellow color represents colocalization. **(B)** Quantification of the colocalization between 6E10 and Tf. **(C)** Quantification of total 6E10 immunoreactivity. **(D)** Quantification of Tf labeling. **(E)** Representative epifluorescence microphotographs of HEKswe cells transfected with GFP control or MT5/GFP plasmids. High power magnification insets represent MT5/GFP or GFP (green), Tf (red) and the merge. Note partial colocalization of MT5/GFP and Tf in the merge panel (yellow color and arrows); arrowheads point out MT5/GFP clusters that do not colocalize with Tf. The nucleus is stained with Hoechst (blue)** (F)** Quantification of the colocalization between Aβ40/EEA1 (early endosomes), Aβ40/Rab11 (recycling endosomes) and Aβ40/Rab7 (late endosomes/lysosomes) in HEKswe after GFP or MT5/GFP transfection. **(G)** Quantification of Aβ40 levels by ELISA assay in the supernatants of HEKswe cells showing a significant increase upon MT5/GFP transfection. The increase in Aβ40 levels was prevented by γ-secretase inhibitor DAPT. Values for **(B–D,F,G)** are the mean ± SEM of four independent experiments. **P < 0.01, ***P < 0.001; Student’s *t* test for **(B–D,F)**. *P < 0.05, ANOVA followed by *post hoc* Fisher’s LSD test for **(G)**. OD, optical density; A.U., arbitrary units.

The above data pose an intriguing scenario where decreases in N-terminal and C-terminal APP fragments would not be necessarily linked to changes in β- and γ-secretase activities. One possibility is that MT5-MMP affects the intracellular distribution/trafficking of APP, as previously hypothesized (Baranger et al., 2016a). To test this idea, we overexpressed MT5-MMP in HEKswe cells that express an *APP* with the Swedish mutation also present in 5xFAD mice, and that have been previously used as a cell model to study APP trafficking (Kaether et al., 2006b). Following transient transfection of the MT5-MMP/GFP (MT5) fusion protein in HEKswe cells, we combined 6E10^+^ immunostaining and Alexa Fluor^®^ 647-Tf pulse labeling for 30 min. In these conditions, Tf mainly labels early endosomes, considered as important loci of β- and γ-secretase activity, and hence of Aβ production (Rajendran and Annaert, 2012; Wang et al., 2014). MT5 expression decreased the levels of 6E10 immunoreactivity in HEKswe cells (Figures 7A,C). On the contrary, 6E10 colocalization with Tf in MT5 expressing cells increased by 82%, compared to GFP (Figures 7A,B). MT5 expression did not modify the content of endosomes with respect to GFP transfected cells (Figures 7A,D). Clearly, MT5 partially colocalized with early endosomes, as revealed by double immunofluorescence with anti-GFP antibody and Alexa Fluor^®^ 647-Tf (Figure 7E).

It is plausible that 6E10 immunostaining in endosomes mainly reflects a pool of C99 and Aβ40, the latter being largely the predominant Aβ species generated in HEKswe cells (Buggia-Prevot et al., 2008). To further identify the distribution of the Aβ40 pool we combined antibodies that recognize the genuine C-terminal end of Aβ40 after C99 processing by γ-secretase, and antibodies against EEA1, Rab11 and Rab7, which are markers of early, recycling and late endosomes, respectively (Figure 7F). MT5 induced a significant 57% increase of Aβ40 colocalization with EEA1 compared to controls, and had no effect on Rab11 or Rab7. Moreover, the supernatants of MT5-transfected cells exhibited a 75% increase of Aβ40 levels, which was prevented by the γ-secretase inhibitor DAPT (Figure 7G).

## Discussion

MT5-MMP is the only member of the MMP family predominantly expressed in the nervous system. Early work highlighted its role in peripheral nervous system (PNS) neuroinflammation (Folgueras et al., 2009), and neuropathic pain after spinal cord lesion (Komori et al., 2004). More recently we demonstrated that MT5-MMP deficiency in the 5xFAD mouse model of AD remarkably ameliorated the outcome of the pathology affecting neocortical and hippocampal functions (Baranger et al., 2016b). The present study extends these data and reveals that MT5-MMP deficiency is also beneficial in the frontal cortex of 5xFAD mice. Thus, preserved memory consistently correlated with a strong reduction of Aβ load, astroglial reactivity and TNF-α levels. Our study also reveals that MT5-MMP deficiency prevented the increase in the levels of soluble N-terminal APP fragments and the neurotoxic transmembrane C99 observed in the frontal cortex of Tg mice. Changes in APP processing in TgMT5^−/−^ mice occurred in the absence of intrinsic proteolytic dysfunctions of β- and γ-secretases. Finally, our data provide evidence that the distribution of APP/Aβ in the endosomal system is altered upon modulation of MT5-MMP expression in a heterologous system, suggesting a new possible mechanism whereby MT5-MMP could drive APP trafficking and its metabolism towards the amyloidogenic pathway.

### MT5-MMP Deficiency Prevents Cognitive Deficits Associated with Frontal Cortex Dysfunctions

In humans and rodents, the frontal cortex is involved in working memory, cognitive flexibility, attention and other cognitive and executive functions that are affected in AD patients (Baddeley et al., 1991; Brugger et al., 1996; Perry and Hodges, 1999). Many reports indicate that age between 4 and 6 months is a transition period where the first behavioral deficits appear in 5xFAD mice, preceding the symptomatic phase of the pathology (Oakley et al., 2006; Jawhar et al., 2012; Girard et al., 2013; Baranger et al., 2016b). The OHM test was conceived to detect dysfunctional cognitive plasticity in the frontal cortex of rodents comparable to that observed in humans with lesions in the same brain area (Del’Guidice et al., 2009; Verin et al., 1993). Like mice with an injured frontal cortex, 4-month old 5xFAD mice showed an impaired ability to solve the OHM delayed response paradigm, which is concomitant with amyloidosis and gliosis (Girard et al., 2013). Here, we demonstrated that frontal cortex-related cognitive deficits of Tg mice were prevented in TgMT5^−/−^, as it was the case for hippocampal-dependent spatial learning and memory (Baranger et al., 2016b). Although spontaneous locomotor activity was similar across genotypes, Tg mice showed reduced anxiety compared to WT mice since they spent more time in the center of the arena. This finding is in agreement with previous observations made in older 5xFAD mice using the same paradigm (Jawhar et al., 2012). Interestingly, the natural anxiety exhibited by rodents in open spaces was somehow recovered in TgMT5^−/−^ mice, whose behavior was closer to that of WT mice. Overall, these data reinforce the idea of an early impairment of executive functions in AD and highlight the role of the frontal cortex in these cognitive processes. Our findings, also stress the influence of MT5-MMP in the neurophysiological mechanisms underlying different brain functions (e.g., working memory, cognitive flexibility and long-term spatial memory), as well as the efficacy of MT5-MMP modulation in improving such functions.

### Improved Learning Deficits Correlates with the Amelioration of Pathological Stigmata in TgMT5^−/−^

The striking reduction in amyloid burden and astrogliosis observed in the frontal cortex of TgMT5^−/−^ mice was also observed in the neocortex and hippocampus (Baranger et al., 2016b), supporting the idea that cognitive dysfunction is tightly associated with biochemical and neuropathological hallmarks present at specific spatio-temporal settings. Such structural/functional correlation may be important for the development of therapeutic strategies that target brain region-associated behaviors. MT5-MMP deficiency reduced astrocyte reactivity and GFAP levels, but microglial reactivity was unaffected, in clear contrast with the reductions that affect both cell types in the neocortex and hippocampi of TgMT5^−/−^ mice compared to Tg (Baranger et al., 2016b). It is noteworthy that increased Iba1 immunoreactivity may not imply *per se* a detrimental type I-like activation of microglia. On the contrary, in this early stage of the pathology, an alternative mode of microglial activation (i.e., type II) could contribute to keep amyloid plaques in a compact rather than a diffuse more pathogenic phenotype, as recently suggested in another AD mouse model (Yuan et al., 2016). Diffuse amyloid plaques have been also correlated with deficient synaptic plasticity in the cortex of 5xFAD mice (Crouzin et al., 2013). These data are consistent with the observation that diffuse/compact amyloid plaque ratio was higher in frontal cortices of Tg compared to TgMT5^−/−^. Another regional difference in TgMT5^−/−^ mice concerns the stable levels of IL-1β in the frontal cortex, in contrast with the reductions observed in the neocortex and hippocampus (Baranger et al., 2016b). Alternatively, TNF-α levels were downregulated in the frontal cortex of TgMT5^−/−^ mice. Along this line, MT5-MMP deficiency has also been linked with deficient inflammatory response to TNF-α in the mouse PNS (Folgueras et al., 2009). Together, these data reinforce the idea that MT5-MMP may influence neuroinflammation in a cytokine- and region-dependent manner. Although synaptic/neuronal markers were preserved, this observation does not preclude the existence of more subtle structural (e.g., shape/size of dendritic spines) or electrophysiological modifications that could account for changes in learning behavior. In line with this idea, preserved synaptic markers in the hippocampus of 4-month old 5xFAD mice (Oakley et al., 2006), are concomitant with impaired LTP and spatial learning in these mice (Kimura and Ohno, 2009; Baranger et al., 2016b; Tang et al., 2016).

### Changes in APP N-terminal and CTFs Occur without Alterations of Secretase Activity in TgMT5^−/−^ Mice

Strong reductions of Aβ load suggested the possibility of altered APP processing upon MT5-MMP deficiency, supported by reduced levels of sAPPα and sAPPβ in bigenic mice without changes of canonical APP. In line with previous findings in the neocortex and hippocampus (Baranger et al., 2016b), sAPP95 levels were reduced in TgMT5^−/−^ brains. This ectodomain fragment likely results from APP processing by MT5-MMP ~100 aminoacids upstream the β-site (between Asn^504^ and Met^505^), as first described *in vitro* (Ahmad et al., 2006), and also reported in the mouse brain below 98 kDa, using in this case an antibody raised against the neoepitope generated by MT5-MMP (Willem et al., 2015). Altogether, these data support the idea that neural APP is an *in vivo* substrate of MT5-MMP. The significant reductions of sAPPβ and its complementary membrane-bound C99 suggested a possible decrease of BACE-1 activity upon MT5-MMP deficiency, which would be consistent with lowered amyloidosis exhibited by TgMT5^−/−^ mice. However, this hypothesis was infirmed by the stability of BACE-1 activity across genotypes in *ex vivo* enzymatic assays using a synthetic substrate, indicating eventually that MT5-MMP deficiency did not affect BACE-1 activity *per se*. We reached the same conclusion with γ-secretase, as the autocatalytic activity of PS1 and the processing of the N-cadherin CTF1 of 37 kDa were not altered by MT5-MMP deficiency. Overall these data are in agreement with our previous work in bigenic TgMT5^−/−^ mice (Baranger et al., 2016b) and with the reported normal processing of Notch (another γ-secretase substrate) in MT5-MMP deficient mice in a non-AD context (Porlan et al., 2014). C99 levels were sharply lowered in TgMT5^−/−^ frontal cortices, in clear contrast with the stability of C83 levels. This is a key observation because C99 has been reported to be neurotoxic on its own (Lauritzen et al., 2012) and its accumulation precedes that of Aβ in the brain of 5xFAD (Py et al., 2014) and 3xTg (Lauritzen et al., 2012) mice. In our conditions, C99 accumulation in the frontal cortex of 4-month old Tg mice is concomitant with higher levels of putative Aβ trimers (~12 kDa), considered as prominent neurotoxic Aβ assemblies (Townsend et al., 2006; Larson and Lesné, 2012). Alternatively, the size of this 6E10^+^ band is compatible with the anticipated size of the recently reported N-terminally elongated Aβ synaptotoxic fragment Aη−α generated in murine and human neurons by combined η-secretase (i.e., MT5-MMP) and α-secretase cleavage, and detected with an antibody raised against the neoepitope generated by η-secretase at Asn^504^-Met^505^ (Willem et al., 2015). It is noteworthy that in this study, the brains of APPPS1-21 transgenic mice exhibited hardly detectable levels of Aη−α, unlike Aη−β resulting from combined action of η- and β-secretase. According to these authors, these data would be consistent with the enhanced BACE-1 affinity for the APP Swedish mutation carried by APPPS1-21. Considering that 5xFAD mice also carry the APP Swedish mutation, it would be plausible that the Aη−α fragment accounts for minor immunoreactive signal of our ~12 kDa 6E10^+^ band relative to other putative Aβ 6E10^+^ assemblies. This clearly excludes the Aη−β fragment, which lacks the 6E10 epitope. Further investigations using *ad hoc* tools, including antibodies that recognize the Aη−α N-terminal neoepitope, will be necessary to determine whether this low molecular weight band represents a mix of neurotoxic Aη−α and Aβ assemblies (e.g., trimers), whose reduction is in any case consistent with the amelioration of the pathological outcome in TgMT5^−/−^ mice.

### MT5-MMP Promotes APP Trafficking in the Endosomal Compartment

Trafficking of APP between the plasma membrane and the intracellular compartments is increasingly considered as a pivotal process for the understanding of amyloidogenesis and hence the pathophysiology of AD (Rajendran and Annaert, 2012). Extensive experimental evidence supports the view that APP not cleaved by γ-secretase at the plasma membrane is rapidly internalized and processed into C99 by BACE-1 in endosomes. The latter provide the acidic microenvironment for optimal BACE-1 function (Haass et al., 2012; Toh and Gleeson, 2016). In these conditions, it is likely that 6E10^+^ immunostaining detected in early endosomes mainly reveals the content of C99 and/or Aβ, as previously suggested (Kaether et al., 2006b). The finding that Aβ40 levels are also upregulated in these organelles supports the idea that both C99 and Aβ coexist in the same compartment, and implicitly, the same holds true for β- and γ-secretase. The precise subcellular localization of β- and γ-secretases is still an open debate, but growing evidence indicates that both may be present in early endosomes (Rajendran and Annaert, 2012; Toh and Gleeson, 2016). Importantly, our data strongly suggest that after internalization MT5-MMP and APP converge towards early endosomes, in agreement with data reported for APP in various cell types (Rajendran and Annaert, 2012; Toh and Gleeson, 2016) and with one report on MT5-MMP in native HEK cells (Wang et al., 2004). Thus, early endosomes could be a privileged subcellular locus of the pro-amyloidogenic interactions between MT5-MMP and APP we described earlier (Baranger et al., 2016b). In the context of MT5-MMP internalization, MT5-MMP deficiency could have a higher impact on APP processing by BACE-1 than by α-secretase, considering their preferential endosomal and plasma membrane localization, respectively. It remains to be investigated whether this might contribute to the differences between C83 and C99 levels in TgMT5^−/−^ mice. In pace with the importance of proteolytic enzymes in APP trafficking and amyloidogenesis, another APP-interacting metalloproteinase, ADAM30, has been shown to promote APP sorting to lysosomes and degradation (Letronne et al., 2016).

In conclusion, the present work provides additional evidence that MT5-MMP contributes to the pathophysiology of AD, and that the underlying mechanisms include, at least in part, the ability of the MMP to promote APP trafficking into amyloidogenic pathways that remain to be thoroughly investigated. The idea that interfering with MT5-MMP activity and/or interactions improves the pathological outcome, supports further validation of this proteinase as a novel promising AD target.

## Author Contributions

KB, AEB, SDG, J-MP, LG-G, WE, AB, EC, DS, CB, KM performed experiments, collected, analyzed and interpreted data. SFL, FSR, FC and MK contributed to designing the work, to interpret data and to critical revision of the manuscript. KB and SR designed the concept of the experiments, interpreted the data and wrote the manuscript. SR supervised the project.

## Conflict of Interest Statement

The authors declare that the research was conducted in the absence of any commercial or financial relationships that could be construed as a potential conflict of interest.
